# Quantum transport of two-species Dirac fermions in dual-gated three-dimensional topological insulators

**DOI:** 10.1038/ncomms11434

**Published:** 2016-05-04

**Authors:** Yang Xu, Ireneusz Miotkowski, Yong P. Chen

**Affiliations:** 1 Department of Physics and Astronomy, Purdue University, West Lafayette, Indiana 47907, USA; 2 Birck Nanotechnology Center, Purdue University, West Lafayette, Indiana 47907, USA; 3 School of Electrical and Computer Engineering, Purdue University, West Lafayette, Indiana 47907, USA

## Abstract

Topological insulators are a novel class of quantum matter with a gapped insulating bulk, yet gapless spin-helical Dirac fermion conducting surface states. Here, we report local and non-local electrical and magneto transport measurements in dual-gated BiSbTeSe_2_ thin film topological insulator devices, with conduction dominated by the spatially separated top and bottom surfaces, each hosting a single species of Dirac fermions with independent gate control over the carrier type and density. We observe many intriguing quantum transport phenomena in such a fully tunable two-species topological Dirac gas, including a zero-magnetic-field minimum conductivity close to twice the conductance quantum at the double Dirac point, a series of ambipolar two-component half-integer Dirac quantum Hall states and an electron-hole total filling factor zero state (with a zero-Hall plateau), exhibiting dissipationless (chiral) and dissipative (non-chiral) edge conduction, respectively. Such a system paves the way to explore rich physics, ranging from topological magnetoelectric effects to exciton condensation.

## Abstract (web summary)

Novel physics of topological aspects are obscured due to lack of effective way to manipulate topological particles. Here, Xu *et al*. demonstrate independent control of Dirac fermions on top and bottom surfaces of BiSbTeSe_2_ flakes by dual-gating, which suggests a way to manipulate exotic particles.

## Graphical abstract

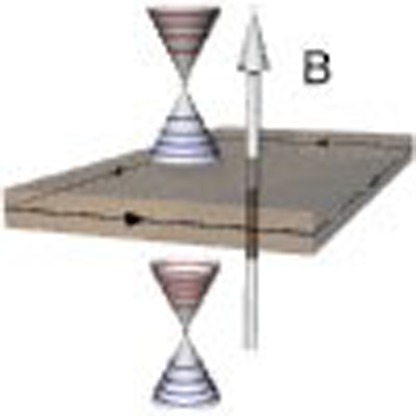

A three-dimensional (3D) topological insulator (TI) is characterized by an insulating bulk band gap and gapless conducting topological surface states (TSS) of spin-helical massless two-dimensional (2D) Dirac fermions^1^,^2^. Such surface states are topologically non-trivial and protected by time-reversal symmetry, thus immune to back scattering. The potential novel physics offered by this system, such as topological magnetoelectric (TME) effects^3^,^4^, Majorana fermions^5^ and effective magnetic monopoles^6^, has drawn intense interest. One of the most iconic transport signatures for 2D Dirac electronic systems is the half-integer quantum Hall effect (QHE) in a perpendicular magnetic field (*B*), as first observed in graphene^7^,^8^ and later also studied in HgTe^9^,^10^. The Landau levels (LLs) of 2D Dirac fermions have energies *E*_N_=sgn(*N*)*v*_F_(*2*e*Bħ*|*N*|)^1/2^, where sgn is the sign function, *N* is the LL index (positive for electrons and negative for holes), *v*_F_ is the Fermi velocity, *e* is the elemental charge and *ħ* is the Plank's constant *h* divided by 2*π*. The zeroth LL at *E*_0_=0 is equally shared between electrons and holes, giving rise to the half-integer shift in the quantized Hall conductivity *σ*_xy_=*g*(*N*+1/2)*e*^2^/*h*, where *g* is the number of degenerate species of Dirac fermions (for example, *g*=4 for graphene, and *g*=1 for TSS with a single Dirac cone). This 1/2 can also be related to the Berry-phase due to the spin or pseudospin locking to the momentum of Dirac fermions^7^,^8^,^9^,^10^.

In most commonly studied TI materials such as Bi_2_Se_3_, Bi_2_Te_3_ and other Bi/Sb-based chalcogenides, it is often challenging to observe characteristic TSS transport (particularly QHE) due to bulk conduction caused by unintentional impurity doping. Only very recently has well-developed QHE arising from TSS been observed in exfoliated flakes from BiSbTeSe_2_ (BSTS) single crystals^11^ and molecular beam epitaxy grown (Bi_1−x_Sb_x_)_2_Te_3_ or Bi_2_Se_3_ thin films^12^,^13^. In this work, we fabricate dual-gated^14^,^15^,^16^ TI devices from exfoliated BSTS thin flakes with undetectable bulk carrier density and conduction at low temperature^11^. Such a dual-gating structure is also promising for exploring exciton condensation proposed for TIs^17^ and topological quantum phase transitions induced by displacement electric field^18^.

In our dual-gated BSTS devices, the independent, ambipolar gating of parallel-conducting top and bottom surfaces realize two independently controlled species of 2D Dirac fermions, allowing us to investigate such interesting transport phenomena as the minimum conductivity of TSS at Dirac point (DP), and two-species (two-component) Dirac fermion QHE of electron+electron, electron+hole and hole+hole types, involving various combinations of top and bottom surface half-integer filling factors *ν*_t_ and *ν*_b_, respectively. When (*ν*_t_, *ν*_b_)=(−1/2, 1/2) or (1/2, −1/2), there's an intriguing *ν*=0 state characterized by zero-Hall plateau and a large longitudinal resistance peak^11^,^12^, attributed to the formation of dissipative and non-chiral edge states. We also perform non-local transport measurements and compare them with the normal local measurements in our dual-gated 3D TI devices in the quantum Hall (QH) regime to probe the nature of edge-state transport for both standard QH states and the novel *ν*=0 dissipative QH-like state. We further demonstrate that the dissipative edge states at *ν*=0 have temperature-independent conductance, revealing that the transport in such a quasi-one-dimensional (1D) dissipative metallic edge channel could evade standard localization.

## Results

### Transport properties at zero and low magnetic field

**Figure 1:**
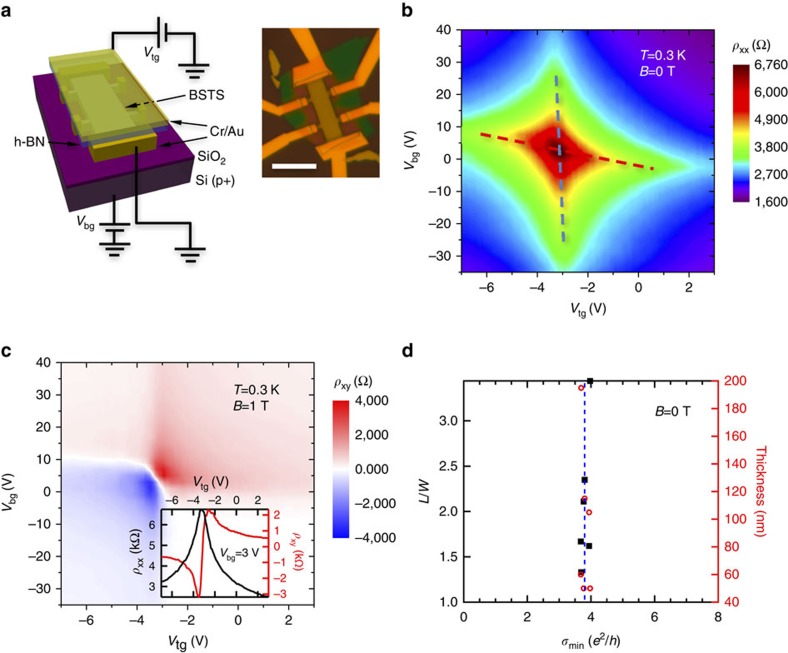
Device configuration and dual-gated field effect at zero and low magnetic field. (**a**) Device schematic. Inset is an optical microscope image of a typical dual-gated BSTS device before depositing the top-gate metal; Scale bar, 10 μm. (**b**,**c**) show 2D maps of *ρ*_xx_ at *B*=0 T and *ρ*_xy_ at *B*=1 T as functions of *V*_tg_ and *V*_bg_ on sample A. The blue (red) dashed lines in **b** are guides to the eye for the top (bottom) surface DP. The 2D map is generated by data measured from *V*_tg_-sweeps at a series of *V*_bg_ values, with one example at *V*_bg_=3 V shown in the inset of **c**. (**d**) Zero-magnetic-field minimum conductivity *σ*_min_ (bottom axis) measured in six dual-gated samples at low temperature (<2 K) plotted as a function of the sample thickness (data in circles) and 2D aspect ratio (*L*/*W*, data in squares). The vertical dashed line indicates 3.8*e*^2^/*h*.

Qualitatively, similar data are measured in multiple samples, while results from a typical sample A (channel length *L*=9.4 μm, width *W*=4.0 μm, with ∼100 nm-thick BSTS and 40 nm-thick h-BN as top-gate dielectric, see schematic in Fig. 1a) are presented below unless otherwise noted. The h-BN as a substrate or gate dielectric is known to preserve good electronic properties for graphene, resulting from the atomic flatness and relatively low density of impurities in h-BN^19^. The carrier densities of the top and bottom surface of the BSTS flake are tuned by top-gate voltage *V*_tg_ and back-gate voltage *V*_bg_, respectively.

Figure 1b,c shows the double-gated electric field effect measured at *T*=0.3 K. The longitudinal resistivity *ρ*_xx_ (=*R*_xx_ × *W*/*L*, with *R*_*xx*_ being longitudinal resistance) at magnetic field *B*=0 T (Fig. 1b) and Hall resistivity *ρ*_xy_ (=*R*_xy_, Hall resistance) at *B*=1 T (Fig. 1c) are plotted in colour scale as functions of both top and bottom gate voltages (*V*_tg_ and *V*_bg_). The extracted field effect and Hall mobilities are typically several thousands of cm^2^ V^−1^ s^−1^. A minimum carrier density n^*^ ∼9 × 10^10^ cm^−2^ per surface can be extracted from the maximum Hall coefficient (absolute value) ∼3.5 kΩ T^−1^ (when both surfaces are slightly n-type or p-type) measured in Fig. 1c. A set of exemplary *V*_tg_-sweeps with *V*_bg_=3 V is shown in Fig. 1c inset. By adjusting *V*_tg_ (or *V*_bg_), the device can be gated through a *R*_xx_ peak, identified as the charge-neutrality DP of the top (or bottom) surface, marked by the blue (or red) dashed lines in Fig. 1b. Gating through the DP, the carriers in the corresponding surface change from hole-like to electron-like (that is, ambipolar), as evidenced by Hall measurements (Fig. 1c). The slight deviation of the two lines from being perfectly vertical and horizontal arises from the weak capacitive coupling between the top (bottom) surface and the back (top) gate^16^. The crossing of these two lines corresponds to the double DP (both surfaces tuned to DP), where *ρ*_xx_ (*σ*_xx_=1/*ρ*_xx_) reaches a global maximum (minimum). Within the gate voltage range used, the carriers predominantly come from the TSS and we observe relatively good particle-hole symmetry in the transport properties (for example, the symmetrical appearance of *ρ*_xx_ on both sides of DP in each surface in Fig. 1b and the similar absolute values of the positive and negative maximum Hall coefficient in Fig. 1c).

We have studied six dual-gated BSTS devices with different thicknesses (*t*) and aspect ratios (*L*/*W*). These devices are measured at low temperatures (*T*<2 K) and the results are repeatable after multiple thermal cycles. When both surfaces are tuned to DP, the minimum 2D conductivity *σ*_min_ at *B*=0 T exhibits relatively constant value (3.8±0.1)*e*^2^/*h* for all the devices measured (with the uncertainty representing 90% confidence interval), whose thicknesses range from ∼50 to ∼200 nm and *L/W* range from 1.3 to 3.5 (Fig. 1d). Our observation indicates that the conductivity at the DP for each major surface (top or bottom) is ∼2*e*^2^/*h* (one unit of conductance quantum), within the range of values (2∼5 *e*^2^/*h*) reported by Kim *et al*.^14^ on thin flakes of Bi_2_Se_3_ (∼10 nm). The better consistency over multiple samples in our dual-gated BSTS devices may be attributed to the more insulating bulk (whose conduction is immeasurably small at low temperature) and uniformity of the exfoliated BSTS flakes, which are sandwiched between SiO_2_ and h-BN to achieve better device stability. The minimum conductivity at DP has also been discussed in graphene with considerable interest^20^,^21^,^22^,^23^,^24^,^25^.

The experiments in graphene revealed that the minimum conductivity is strongly affected by carrier-density inhomogeneities (puddles) induced by disorder on or near graphene^24^,^25^, such as the absorbates or charged impurities in the substrates. In 3D TIs, one source of impurities likely relevant to the observed quasi-universal minimum conductivity in our dual-gated BSTS devices could be bulk defects (located near surface)^26^,^27^, such as those revealed in scanning tunnelling microscopy studies^28^.

### Two-component QHE

**Figure 2:**
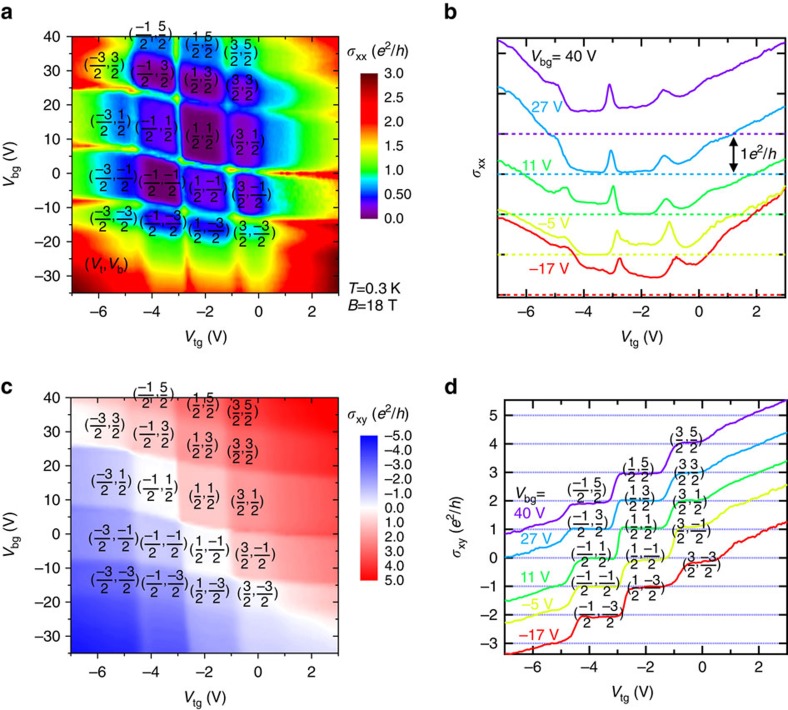
QHE modulated by top and bottom gates. (**a**) *σ*_xx_ and (**c**) *σ*_xy_, shown as 2D colour maps, as functions of *V*_tg_ and *V*_bg_ at *B*=18 T and *T*=0.3 K in the sample ‘A', with representative cuts at 5 different values of *V*_bg_ shown in **b** and **d**. The (*ν*_t_, *ν*_b_) labels (top, bottom) surface filling factors for corresponding quantum Hall states. The *σ*_xx_ curves in **b** are shifted vertically (in consecutive step of *e*^2^*/h*) for clarity (the corresponding zero *σ*_xx_ levels are indicated by the same-coloured horizontal dashed lines).

For the rest of the paper, we focus on the transport phenomena in the QH regime under a high magnetic field *B* perpendicular to the top and bottom surfaces. Figure 2a,c shows in colour scales the longitudinal conductivity *σ*_xx_ (=*ρ*_xx_/(*ρ*_xx_^2^+*ρ*_xy_^2^)) and Hall conductivity *σ*_xy_ (=*ρ*_xy_/(*ρ*_xx_^2^+*ρ*_xy_^2^)) for Sample A as functions of *V*_*tg*_ and *V*_*bg*_ at *B*=18 T and *T*=0.3 K. The colour plots in Fig. 2a,c divide the (*V*_tg_, *V*_bg_) plane into a series of approximate parallelograms, centred around well-developed or developing QH states with vanishing or minimal *σ*_xx_ (Fig. 2b) and quantized *σ*_xy_ in integer units of *e*^2^/*h* (Fig. 2d). These QH parallelograms are bounded by approximately (but slightly tilted) vertical and horizontal lines, which represent the top and bottom surface LLs, respectively. By increasing (decreasing) either *V*_tg_ or *V*_bg_ to fill (exhaust) one LL on the top or bottom surface, *σ*_xy_ increases (decreases) by *e*^2^/*h*, taking consecutive quantized values of *νe*^2^/*h*, where integer *ν=ν*_t_+*ν*_b_=*N*_t_+*N*_b_+1. The *N*_t(b)_ is the corresponding top (bottom) surface LL integer index that can be adjusted by top (back) gate to be of either Dirac electrons or holes. In Fig. 2d, different fixed *V*_bg_ values (from −17 to 40 V) set *ν*_b_ around consecutive half integers −3/2, −1/2, 1/2, 3/2 and 5/2 (such that the bottom surface contributes *σ*_xy_^b^=*ν*_b_*e*^2^/*h* to the total *σ*_xy_), explaining the vertical shift of *e*^2^/*h* at QH plateaux of consecutive *V*_tg_-sweeps.

**Figure 3:**
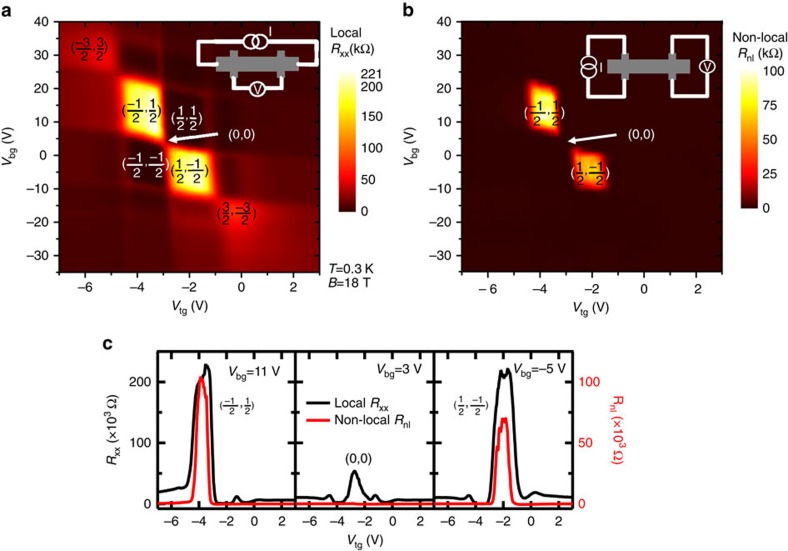
Local and non-local resistance in dual-gated TI in high magnetic field. (**a**) Local resistance *R*_xx_ and (**b**) non-local resistance *R*_nl_ measured in sample A as functions of *V*_tg_ and *V*_bg_ at *B*=18 T and *T*=0.3 K, with insets showing the measurement setup schematics. (**c**) A few representative cuts of **a** and **b** at different values of *V*_bg_. Filing factors for the local *R*_xx_ peaks are labeled in each sub-panel.

It is also notable that in Fig. 2, there are a few states with zero-quantized Hall conductivity (*σ*_xy_=0, manifesting as white regions in Fig. 2c, separating the electron-dominated regions in red and the hole-dominated regions in blue) and non-zero *σ*_xx_ minimum, marked by equal and opposite half-integer values of *ν*_t_ and *ν*_b_ thus total *ν*=0, for example (*ν*_t_, *ν*_b_)=(−1/2, 1/2), (1/2, −1/2) and (3/2, −3/2). These states with total *ν*=0, exhibiting zero-Hall plateaux (see also Fig. 2d), have non-zero *σ*_xx_ minimum (Fig. 2a,b) but very large *R*_xx_ maximum (see next, Fig. 3).

### Non-local transport at *ν*=0 states

To further characterize the observed QH and *ν*=0 states, we have performed non-local transport measurements of *R*_nl_ (=*V*_nl_/*I*, *I* is the current and *V*_nl_ is the non-local voltage, see the schematic measurement setup in the inset of Fig. 3b) as functions of *V*_tg_ and *V*_bg_ at *B*=18 T and *T*=0.3 K and compared the results with the standard (local) measurements of the longitudinal resistance *R*_xx_ (Fig. 3a). It is intriguing that unlike other QH states typified by a zero or minimum in *R*_xx_, the states with *ν*=*ν*_t_+*ν*_b_=0 (labelled by (*ν*_t_, *ν*_b_) in Fig. 3a with *ν*_t_=−*ν*_b_ =±1/2 or ±3/2) are accompanied by a *R*_xx_ maximum. The best-developed *ν*=0 states are those at (*ν*_t_, *ν*_b_)=(−1/2, 1/2) or (1/2, −1/2), where *R*_xx_ reaches ∼220 kΩ (*ρ*_xx_ ∼100 kΩ), exceeding the resistance quantum (*h*/*e*^2^=∼25.8 kΩ) by an order of magnitude. The non-local *R*_nl_ also becomes very large (∼100 kΩ) and the similar order of magnitude as *R*_xx_ at these two *ν*=0 states, while negligibly small at other (*ν*_t_, *ν*_b_) QH states (see Fig. 3b and also the representative cuts in Fig. 3c).

The simultaneously large local and non-local resistance at *ν*=0 states in the QH regime has been reported in other 2D electron-hole systems^29^,^30^ and understood in a picture of dissipative edge channels. We emphasize that the pronounced *R*_nl_ signal cannot be explained from *R*_xx_ by a classical Ohmic non-local resistance from the stray current connecting the remote leads. Such a contribution (=∼*ρ*_xx_*e*^−*πL*/*W*^) would decay exponentially with *L*/*W* (=2.4 in our case), and be three orders of magnitude smaller than the local *R*_xx_ (which is the case at *B*=0 T, Supplementary Fig. 1). As another comparison, the middle panel of Fig. 3c shows the cuts in Fig. 3a,b at *V*_bg_=3 V, crossing the double-DP (also zeroth LL) of both top and bottom surfaces at (*ν*_t_, *ν*_b_)=(0, 0), where we observe a relatively large peak in *R*_xx_ but significantly smaller *R*_nl_. Such a result is consistent with the ‘extended' state transport (at the center of zeroth LL) as the current flows through the bulk of the 2D surface.

From the colour plots in Figs 2 and 3, the parallelogram centred around (*ν*_t_, *ν*_b_)=(−1/2, 1/2) state is enclosed by boundaries representing *N*_t_=0 and −1, *N*_b_=0 and 1 LLs. Similarly, the (*ν*_t_, *ν*_b_)=(1/2, −1/2) state is bound by *N*_t_=0 and 1, *N*_b_=0 and −1 LLs. We conclude that such a *ν*=0 state can exist when the potential difference *V* between top and bottom surfaces (equivalently the energy separation between top and bottom surface DPs) is in the range of 0<|*V|*<2*E*_0−1_ (≅2 × 50 meV at *B*=18 T, where *E*_0−1_ is the 0−1 LL separation of TSS Dirac fermions^11^). The large energy scale of *E*_0−1_ can help make the *ν*=0 and *ν*=±1 QH states observable at significantly elevated temperatures as demonstrated below.

### Temperature dependence of the *ν*=0 and ±1 states

**Figure 4:**
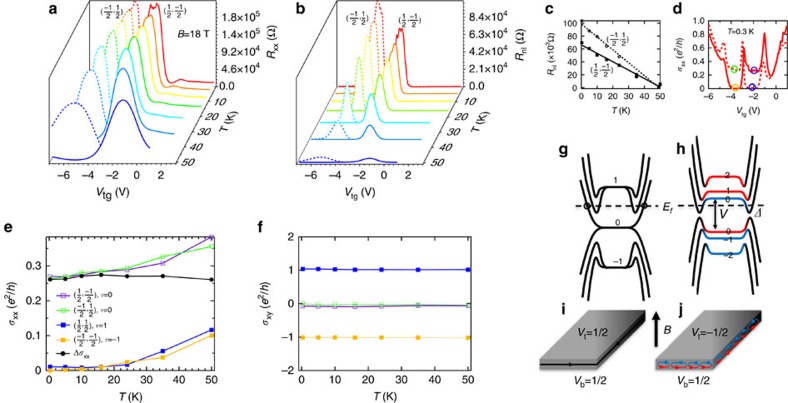
Temperature dependence and illustrative schematics of the QHE and *v*=0 state in TI. (**a**) *R*_xx_ and (**b**) *R*_nl_ measured in sample ‘A' as functions of *V*_tg_ for different temperatures at *B*=18 T, where *V*_bg_ is chosen to set *ν*_b_ at 1/2 (dashed lines) and −1/2 (solid lines), respectively. (**c**) The *R*_nl_ value at (*ν*_t_, *ν*_b_)=(−1/2, 1/2) and (1/2, −1/2) shows approximately linear dependence on temperature. (**d**) *σ*_xx_ versus *V*_tg_ (with the same two values of *V*_bg_ chosen in a and b) at *T*=0.3 K as an example, with each highlighted circle corresponding to a state in **e** plotted with corresponding coloured symbols. (**e**) *σ*_xx_ and (**f**) *σ*_xy_ of *ν*=+1, −1 and 0 states as functions of temperature. In **e**, we also plot *Δσ*_xx_ (difference between averaged *ν*=0 states' *σ*_xx_ and averaged *ν*=±1 states' *σ*_xx_), which barely changes with *T*. (**g**,**h**) Schematics of surface band structure (energy spectrum) in high magnetic field, showing LLs from top and bottom surfaces (blue and red) in the middle of the sample transitioning into side surface sub-bands at sample edge, and (**i**,**j**) edge states in a slab-shaped sample for *ν*=1 and *ν*=0 states. The dashed line indicates a representative Fermi level *E*_f_ and circles in **g** label chiral edge modes.

We have studied the temperature (*T*) dependence of the QHE and *ν*=0 states from 0.3 K to 50 K at *B*=18 T (Fig. 4). At each temperature, the bottom surface density is tuned by *V*_bg_ to set *ν*_b_ near 1/2 (dashed lines) or −1/2 (solid lines), and the peaks in local *R*_xx_ and non-local *R*_nl_ corresponds to the (*ν*_t_, *ν*_b_)=(−1/2, 1/2) or (1/2, −1/2), respectively (Fig. 4a,b, detailed raw data are shown in Supplementary Fig. 2). The *R*_xx_ peaks (>∼150 kΩ) are seen to be more robust up to the highest temperature (*T*=50 K) measured, while *R*_nl_ peaks decrease rapidly (approximately linearly in *T*, shown in Fig. 4c) with increasing *T* and is nearly suppressed above 50 K. We also show the *T*-dependence of *σ*_xx_ and *σ*_xy_ at (*ν*_t_, *ν*_b_)=(−1/2, 1/2), (1/2, −1/2), (1/2, 1/2) and (−1/2, −1/2) in Fig. 4e,f. The *σ*_xy_ maintains good quantization at *νe*^2^/*h* (*ν*=0, ±1) up to *T*=50 K, while *σ*_xx_ increases with *T* (the gate-dependent *σ*_xx_ and *σ*_xy_ traces at different temperatures are shown in Supplementary Fig. 3). The *σ*_xx_ for *ν*=±1 states is found to show thermally activated behaviour at high temperatures^11^, where the finite *σ*_xx_ is attributed to the thermally excited 2D surface or 3D bulk carriers. Such carriers can shunt the edge-state transport and suppress the non-local *R*_nl_ response at high *T* (ref. 29). We also note that the *σ*_xx_ versus *T* curves for *ν*=0 and *ν*=±1 states follow the similar trend and have approximately constant separation. We find the averaged separation *Δσ*_xx_=1/2 × (*σ*_xx_(−1/2, 1/2)+*σ*_xx_(1/2, −1/2)−*σ*_xx_(1/2, 1/2)−*σ*_xx_(−1/2, −1/2)) to be largely *T*-independent with a value of (0.27±0.01)*e*^2^/*h*, which we attribute to the conductivity of the quasi-1D dissipative edge channel.

## Discussions

In our measurement setup, the contacts connect to the top, bottom and side surfaces, all of which are probed simultaneously. The side surface only experiences an in-plane field and can be viewed as a quasi-1D domain boundary that separates the top and bottom surfaces with *B* pointing outward and inward, respectively, thus can support QH edge states^31^. When the top and bottom surfaces are doped to the same carrier type (either n or p), the corresponding QH edge states (on the side surface) would have the same chirality and give the observed total *σ*_xy_=*νe*^2^/*h*=(*ν*_t_+*ν*_b_)*e*^2^/*h*, restricted to integer multiples of *e*^2^/*h*. When the two surfaces have opposite carrier types but one of the them dominates, well-defined QH states with *ν*=*ν*_t_+*ν*_b_ may still be observed, such as the (−1/2, 3/2) state with *σ*_xy_=(−1/2+3/2)*e*^2^/*h*=*e*^2^/*h* and vanishing *ρ*_xx_. Previous studies in InAs/(AlSb)/GaSb heterostructure-based electron-hole systems^32^,^33^ also revealed QH effect with *R*_xy_=*h*/(*νe*^2^)=*h*/(*ν*_e_−*ν*_h_)*e*^2^ (*ν*_e_ and *ν*_h_ are electron and hole-filling factors, both are positive integers) and vanishing *R*_xx_ when the AlSb barrier (separating electron and hole gases) is sufficiently thin to enable electron-hole hybridization. Despite the phenomenological similarities, our QH system is distinctive in the sense that the spatially separated electrons and holes residing on the top and bottom surfaces have *half*-integer filling factors, and the hybridization only happens at the side surface.

We show the schematic energy spectrum when the two surfaces are degenerate with *V*=0 (refs 34, 35, 36) in Fig. 4g, which depicts the Fermi energy *E*_f_ inside the 0−1 LL gap and corresponds to the (1/2, 1/2) QH state. For a relatively thick sample such as ours (∼100 nm>magnetic length *l*_B_=(*ħ*/*eB*)^1/2^≅6 nm at *B*=18 T); however, it has been suggested that even in the presence of well-quantized LLs, a standard TI Hall measurement would exhibit deviations from perfectly quantized values due to conduction through the side surfaces^31^,^34^,^35^,^36^. On the other hand, it has also been suggested that when net chiral modes exist (Fig. 4g,i show one such net chiral mode), the QH effect may be restored by the local equilibrium between non-chiral edge modes^37^, possibly explaining the good quantization in *σ*_xy_ and vanishing *ρ*_xx_ (also *R*_nl_) observed in our experiments.

For the (*ν*_t_, *ν*_b_)= (−1/2, 1/2) or (1/2, −1/2) state, the carrier density on the top and bottom surfaces are opposite. Since *E*_f_ is within the LL gap on both the surfaces, the finite residual *σ*_xx_ and large *R*_nl_ we observed are indicative of dissipative edge transport. We show a schematic energy spectrum^38^ of this *ν*=0 state with *V* slightly smaller than *E*_0−1_ in Fig. 4h, where the Fermi level *E*_f_ resides between the *N*_t_=−1 and 0 LL of top surface (marked in blue), thus *ν*_t_=−1/2, and also between the *N*_b_=0 and 1 LL of bottom surface, thus *ν*_b_=1/2. Overall, such energy spectrum represents a (*ν*_t_, *ν*_b_)=(−1/2, 1/2) and *ν*=0 state. The *E*_f_ crosses an even number (only two shown in this illustrative example in Fig. 4h) of counter-propagating edge modes (arising from sub-bands of the quasi-1D side surface). The disorder can cause scattering and local equilibrium between the counter-propagating modes, giving rise to non-chiral dissipative transport (depicted by a series of conducting loops that can hop between adjacent ones in Fig. 4j) on the side surface with a large and finite resistance. While the energy spectrum (Fig. 4h) is expected to have a gap (*Δ*) near the edge (due to the hybridization between top (marked with blue) and bottom (red) surface zeroth LLs and approximately the finite-size confinement-induced gap ≅*hv*_F_/*t*≅10 meV opened at DP of the side surface^38^), we did not observe a truly insulating state with vanishing *σ*_xx_ and diverging *R*_xx_ (Figs 2a and 4a). This is likely due to the disorder potential (spatial fluctuation of DP^28^) comparable or larger than *Δ* and thus smearing out this gap (effectively *E*_f_ always crosses the non-chiral edge modes). It would be an interesting question for future studies to clarify whether the weak *T*-dependence (at *T*<∼50 K) of the observed conductance (Fig. 4e), similar to the behaviour reported in InAs/GaSb based electron-hole systems^39^, may indicate an absence of localization^40^,^41^,^42^,^43^ in such quasi-1D resistive edge channels.

Several recent theories have pointed out that the *ν*=1/2−1/2=0 state in the TI QH system may bring unique opportunities to realize various novel physics. It has been suggested that both the *ν*=0 state in TI QHE and an analogous quantum anomalous Hall (QAH) state with zero-Hall-conductance plateau in a magnetic-doped TI around the coercive field can be used as platforms to observe the TME effect^44^,^45^, where an electric (magnetic) field induces a co-linear magnetic (electric) polarization with a quantized magnetoelectric polarizability of ±*e*^2^*/*2 *h*. A zero-Hall-plateau state has been recently observed in the QAH case in ultrathin (few-nm-thick) films of Cr_x_(Bi,Sb)_2-x_Te_3_ at low temperature (<1 K) (refs 46, 47). In comparison, our samples have much larger thickness (>∼50 nm, suggested to be preferable for better developed TME effect^45^,^48^), and our *ν*=0 state survives at much higher temperatures (∼50 K). It has also been proposed that excitonic condensation and superfluidity can occur in thin 3D TIs at the *ν*=0 state in QH regime^49^ (in addition to the at-zero *B* field^17^) induced by spontaneous coherence between strongly-interacting top and bottom surfaces. In future studies, much thinner samples are likely needed to investigate the possibility of such exciton superfluidity.

## Methods

### Sample preparation

3D TI single crystals BiSbTeSe_2_ (BSTS) were grown by the vertical Bridgman technique^11^. BSTS flakes (typical thickness ∼50–200 nm) are exfoliated (Scotch tape method) onto highly doped Si (p+) substrates (with 300 nm-thick SiO_2_ coating), and lithographically fabricated into Hall bar-shaped devices with Cr/Au contacts. A thin flake of hexagonal boron nitride (h-BN, typical thickness ∼10–40 nm) is transferred^19^ on the BSTS flake to serve as a top-gate dielectric and a top-gate metal (Cr/Au) is deposited afterwards. The thickness of BSTS and h-BN flakes are measured by atomic force microscopy.

### Transport measurement

Transport measurements are performed with the standard lock-in technique using a low-frequency (<20 Hz) excitation current of 20 nA in a helium-4 variable temperature system (with base temperature down to 1.6 K) or a helium-3 system equipped with magnetic fields (*B*) up to 18 T (down to 0.3 K).

## Additional information

**How to cite this article:** Xu, Y. *et al*. Quantum transport of two-species Dirac fermions in dual-gated three-dimensional topological insulators. *Nat. Commun.* 7:11434 doi: 10.1038/ncomms11434 (2016).

## Supplementary Material

Supplementary Information Supplementary Figures 1-3
