# Supplementary material for: Quantum transport of two-species Dirac fermions in dual-gated three-dimensional topological insulators
Source: Nat Commun. 2016 May 4;7:11434. doi: 10.1038/ncomms11434 (PMC4857391; doi:10.1038/ncomms11434)
Supplement: Supplementary Information — Supplementary Figures 1-3 [file ncomms11434-s1.pdf]

Supplementary Information

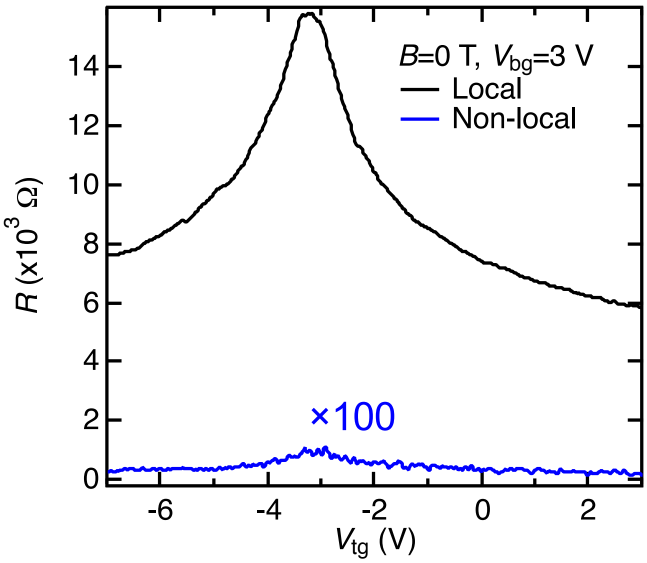

Supplementary Figure 1 | Measured local and nonlocal resistance without magnetic field. Local resistance R_{xx} (a) and non-local resistance R_{nl} (b) as functions of V_{tg} at V_{bg}=3 V and B=0 T, T=0.3 K. The non-local resistance R_{nl} shown here is multiplied by a factor of hundred. The data shown in this supplementary file are all from the same Sample A as that in the main text.

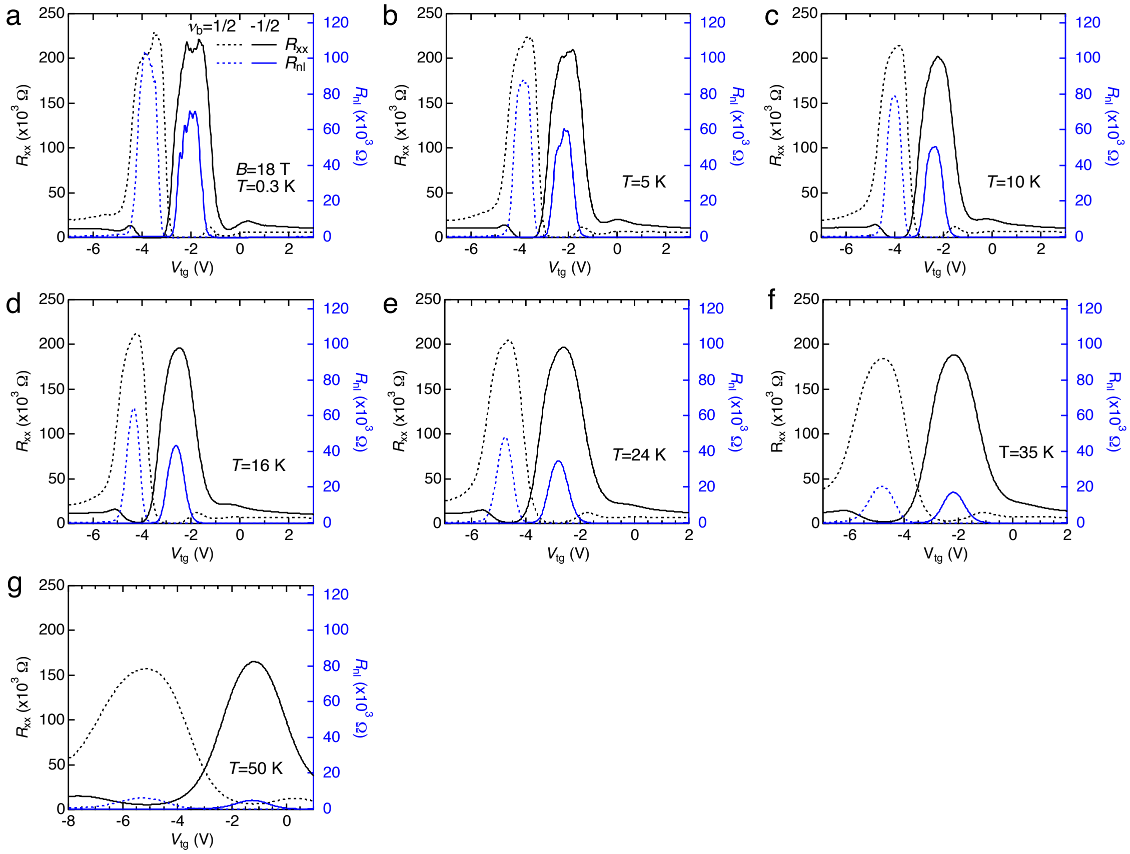

Supplementary Figure 2 | Temperature dependence of the local and nonlocal resistance. (a)-(g) Local resistance R_{xx} and nonlocal resistance R_{nl} versus V_{tg} at fixed bottom surface filling factor ν_b=1/2 (dashed) and -1/2 (solid) measured at B=18 T at different temperatures. At total filling factor ν=0 states, the nonlocal resistance R_{nl} decreases more rapidly with increasing temperature while the local resistance R_{xx} maintains large value up to 50 K.

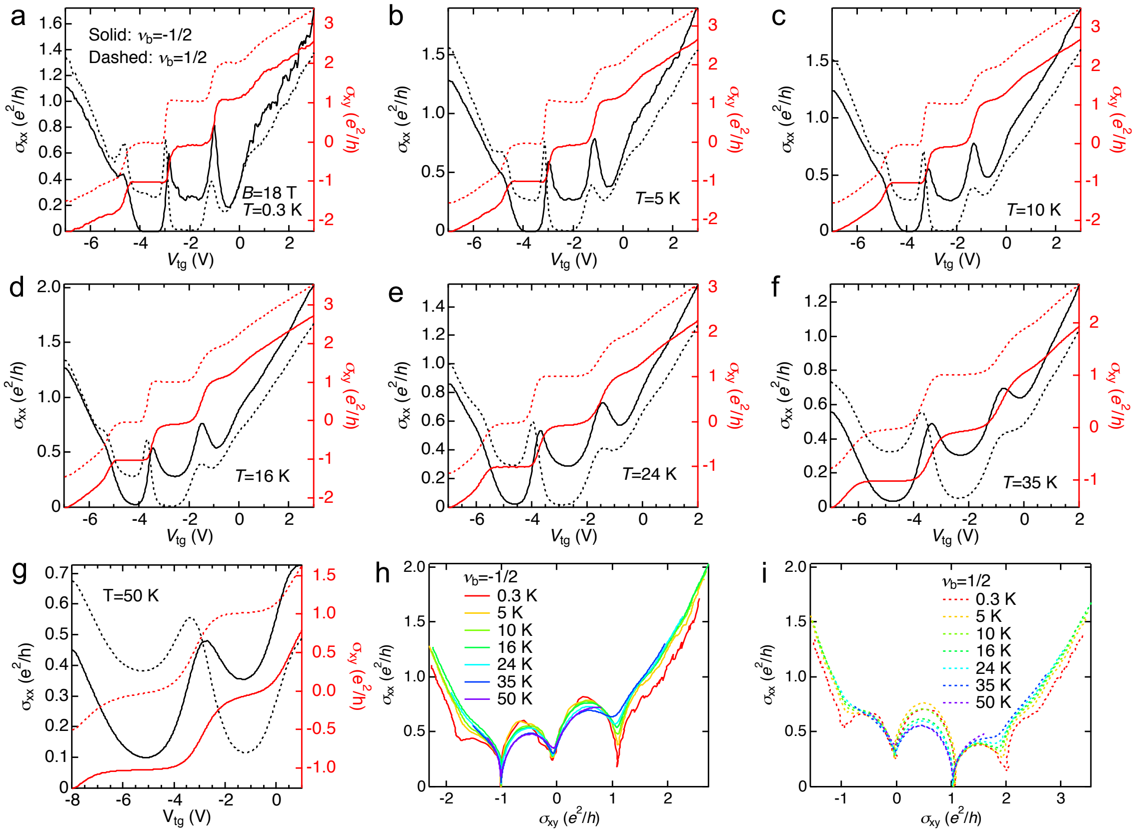

Supplementary Figure 3 | Temperature dependence of the longitudinal and Hall resistivity. (a)-(g) Longitudinal conductivity σ_{xx} and Hall conductivity σ_{xy} versus V_{tg} at fixed bottom surface filling factor ν_b=1/2 (dashed) and -1/2 (solid) measured at B=18 T at different temperatures. (h) and (i) show σ_{xx} versus σ_{xy} at ν_b=-1/2 and 1/2 respectively at different temperatures.
